# Pathogenesis of Nonalcoholic Steatohepatitis and Hormone-Based Therapeutic Approaches

**DOI:** 10.3389/fendo.2018.00485

**Published:** 2018-08-24

**Authors:** Kook Hwan Kim, Myung-Shik Lee

**Affiliations:** ^1^Severance Biomedical Research Institute, Yonsei University College of Medicine, Seoul, South Korea; ^2^Department of Internal Medicine, Yonsei University College of Medicine, Seoul, South Korea

**Keywords:** NAFLD, NASH, steatosis, inflammation, fibrosis, multiple-parallel hit

## Abstract

Non-alcoholic fatty liver disease (NAFLD) is an emerging global health problem and a potential risk factor for type 2 diabetes, cardiovascular disease, and chronic kidney disease. Nonalcoholic steatohepatitis (NASH), an advanced form of NAFLD, is a predisposing factor for development of cirrhosis and hepatocellular carcinoma. The increasing prevalence of NASH emphasizes the need for novel therapeutic approaches. Although therapeutic drugs against NASH are not yet available, fundamental insights into the pathogenesis of NASH have been made during the past few decades. Multiple therapeutic strategies have been developed and are currently being explored in clinical trials or preclinical testing. The pathogenesis of NASH involves multiple intracellular/extracellular events in various cell types in the liver or crosstalk events between the liver and other organs. Here, we review current findings and knowledge regarding the pathogenesis of NASH, focusing on the most recent advances. We also highlight hormone-based therapeutic approaches for treatment of NASH.

## Introduction

The liver is a central metabolic organ that coordinates whole-body energy homeostasis by regulating glucose, lipid, and protein metabolism. The liver is also the main organ of detoxification and processes pharmaceutical products or environmental xenobiotics absorbed from the intestine. Therefore, liver diseases can cause systemic metabolic abnormalities; conversely, the liver is an important target organ of diverse metabolic disorders, which may lead to the development of non-alcoholic fatty liver disease (NAFLD), alcoholic fatty liver disease (AFLD), cirrhosis, and hepatocellular carcinoma (HCC). Among these diseases, NAFLD is one of the most prevalent chronic liver diseases and is an emerging global public health threat. NAFLD affects about 1.8 billion people worldwide with a prevalence of ~20–30% (1). The pathological spectrum of NAFLD ranges from simple steatosis to advanced stages including non-alcoholic steatohepatitis (NASH), hepatic fibrosis, and cirrhosis. The prevalence of simple steatosis with lipid accumulation exceeding 5% of liver weight ranges from 15 to 40% in the general population. Among patients with simple steatosis, 10–20% develop NASH which is defined as steatosis with hepatic inflammation and fibrosis (1). NASH can progress to more severe stages such as cirrhosis and HCC (2). NAFLD/NASH is an emerging risk factor for type 2 diabetes, cardiovascular disease, and chronic kidney disease (3). In particular, NAFLD/NASH is closely associated with several metabolic disorders such as obesity, dyslipidemia, and type 2 diabetes (3). It is estimated that 70–80% of obese or diabetic subjects have NAFLD, with a NASH prevalence of 10–20%. Despite the clinical importance of NAFLD/NASH, therapeutic drugs against these diseases have not yet been developed. However, numerous studies suggest that NASH develops by multiple intracellular/extracellular events in different liver cell types such as hepatocytes, hepatic stellate cells (HSCs), Kupffer cells (resident macrophages in the liver), and infiltrating macrophages (4) and by inter-organ crosstalk between the liver and other tissues including adipose tissue or the intestine (5, 6). This “multiple-parallel hit” model has recently been considered as a more adequate hypothesis to understand the pathogenesis of NASH than the “two-hit” model in which hepatic steatosis, the “first hit,” increases susceptibility to NASH caused by a “second hit” such as oxidative stress and inflammatory cytokines (7). Here, we briefly highlight molecular mechanisms of the pathogenesis of NASH, focusing on recent findings supporting the “multiple-parallel hit” hypothesis. We also describe potential therapeutic strategies based on hormones for treatment of NASH.

## “Multiple-parallel hit” pathogenesis of NASH

**Figure 1:**
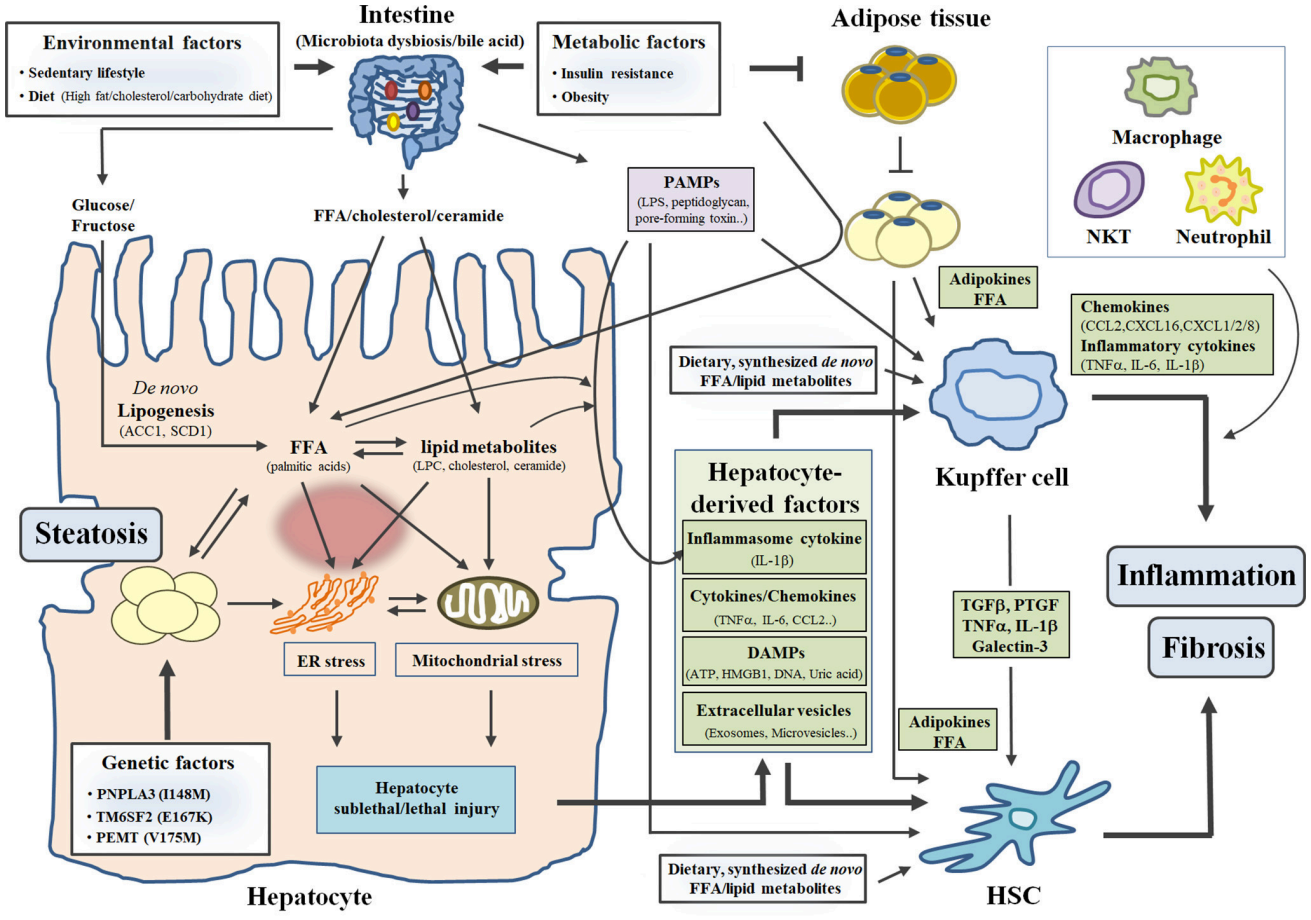
The “mutiple-parallel hit” model in the pathogenesis of NASH. Three factors (environmental, metabolic and genetic factors) contribute to the development and progression of NASH by affecting diverse organs such as the liver, the intestine, and adipose tissue. In particular, excess caloric or inappropriate intake (environmental factor) increases toxic free fatty acid (FFA) and lipid metabolites (LPC, cholesterol and ceramide) in hepatocytes, leading to hepatic steatosis and hepatocyte sublethal/lethal injuries. Subsequently, hepatocyte-derived factors (such as cytokines/chemokines, DAMPs and extracellular vesicles) stimulate inflammatory response in Kupffer cell and fibrotic response in HSC, which leads to the development of inflammation and fibrosis in the liver. FFA and lipid metabolites derived from diets or synthesized *de novo* also activates Kupffer cell and HSC. In addition, insulin resistance and obesity (metabolic factor) influence organ-crosstalk between the intestine/adipose tissue and the liver, contributing to the development and progression of NASH.

Hepatocytes, Kupffer cells/infiltrating macrophages, and HSCs play key roles in hepatic steatosis, inflammation, and fibrosis, respectively. In this section, we discuss intracellular events in individual cells and intercellular crosstalk between different cell types within the liver in the pathogenesis of NASH, focusing on recent advances in the potential role of hepatocytes. We also briefly describe the importance of the adipose tissue-liver axis and intestine-liver axis in the pathogenesis of NASH, emphasizing the most recent findings (Figure 1).

### Hepatic intracellular and intercellular crosstalk and NASH

#### Hepatocyte lipid accumulation

Hepatic steatosis develops by interactions among genetic, metabolic, and environmental factors (8). Sedentary lifestyle and excess caloric or inappropriate intake (high glucose, high fructose, high fat, or high cholesterol diet) are well-known environmental factors associated with hepatic steatosis and NAFLD/NASH. Excess dietary glucose or fructose enters the liver through the bloodstream after uptake in the small intestine and is subsequently utilized for the production of hepatic triacylglycerol (TG) via *de novo* fatty acid synthesis, as well as being a source for hepatic glycogen storage. Dietary fatty acids absorbed in the small intestine also contribute to hepatic TG formation by two different pathways. In the first, free short-chain fatty acids transported into the blood from the small intestine are taken up by the liver via fatty acid transporter [cluster of differentiation 36 (CD36), fatty acid transporter protein (FATP)] together with free fatty acid (FFA) “spillover” generated from lipoprotein lipase (LPL)-mediated chylomicron hydrolysis. In the second pathway, chylomicron TGs containing middle/long-chain fatty acids are hydrolyzed by LPL in peripheral tissues such as skeletal muscle and adipose tissue and consequently become chylomicron remnants, which are taken up by the liver through their receptors [low density lipoprotein receptor (LDLR) and LDL receptor-related protein (LRP)].

In addition to environmental factors, metabolic factors such as insulin resistance, obesity, and dyslipidemia increase susceptibility to hepatic steatosis and NAFLD/NASH (9, 10). In particular, insulin resistance in adipose tissue impairs the ability of insulin to suppress lipolysis; consequently, FFAs released from adipose tissue are transported into the liver. FFAs from adipose tissue contribute to ~60% of hepatic lipid accumulation together with FFA “spillover” (11). Hyperinsulinemia or hyperglycemia, a key feature of insulin resistance, also increases hepatic *de novo* fatty acid synthesis via upregulation of SREBP1 and ChREBP, impairs hepatic β-oxidation, and enhances CD36-mediated fatty acid uptake, thereby contributing to the development of hepatic steatosis (12, 13). Genome-wide association studies suggest that variation in several genes [e.g., *PNPLA3* (patatin-like phospholipase domain-containing protein 3), *TM6SP2* (transmembrane 6 superfamily member 2), and *FADS* (fatty acid desaturase)] related to hepatic lipid metabolism are associated with increased hepatic steatosis in human NAFLD subjects (14–16). A more detailed discussion regarding the contribution of genetic (or epigenetic) factors to the pathogenesis of NASH has been recently reviewed elsewhere (17). Thus, various risk factors contribute to the development of hepatic steatosis and NALFD/NASH by affecting hepatic lipid metabolism through multiple pathways (Figure 1).

#### Hepatocyte oxidative stress, lipotoxicity, and cell death

Excessive lipid accumulation in the liver causes hepatocellular lipotoxicity via cellular and organelle oxidative stresses including endoplasmic reticulum (ER) stress and mitochondrial dysfunction, eventually leading to hepatocyte cell death (Figure 1) (18, 19). In particular, ER stress is implicated in the development and progression of NAFLD (20, 21). When cells are exposed in ER stress, the integrated stress signal mediated by three ER stress sensors (PERK, IRE1α, and ATF6) triggers unfolded protein response (UPR), which serves as a compensatory mechanism to maintain ER homeostasis. However, prolonged or excessive lipotoxic ER stress overwhelms the capacity of UPR and induces hepatic cell death via two main pathways (a mitochondria-dependent intrinsic pathway and a death receptor-mediated extrinsic pathway) (22). Moreover, chronic ER stress increases the production of reactive oxygen species (ROS) and activates the NF-κB or c-Jun N-terminal kinase (JNK) pathway, leading to hepatic inflammation (21). In addition, chronic ER stress results in hepatic lipid accumulation via activation of *de novo* fatty acid synthesis in a manner dependent on ER stress-sensing pathways (23), suggesting that a vicious cycle between ER stress and hepatic steatosis may promote the development and progression of NAFLD/NASH.

The roles of saturated fatty acids and other lipid metabolites [lysophosphatidylcholine (LPC), ceramide, and free cholesterol] as potential mediators of hepatocellular lipotoxicity in NAFLD/NASH have recently emerged (6, 19). Palmitic acid, the most abundant long-chain saturated fatty acid *in vivo*, has been reported to trigger oxidative stress and ER/lysosomal/mitochondrial stresses, causing lipotoxicity-related cell death (24–26). LPC and ceramide have been also reported to act as lipid intermediates mediating the effect of palmitic acid on hepatocellular lipotoxicity (27). In particular, LPC generated from palmitic acid triggers mitochondria-dependent apoptotic machinery via activation of the G-protein-coupled receptor Galpha(i)-JNK pathway (27) or of the CCAAT/enhancer binding homologous protein (CHOP)/JNK pathway (28). Ceramide synthesized via a *de novo* pathway from palmitic acid also causes mitochondrial dysfunction (such as impairment of mitochondrial respiratory chain and increase of mitochondrial membrane permeability), leading to hepatocyte cell death (29). In addition, ceramide generated from tumor necrosis factor alpha (TNFα)-induced activation of the acid sphingomyelinase (ASMase) pathway contributes to hepatocyte apoptosis (30). Another lipid metabolite, free cholesterol, can also induce hepatocyte necrosis/apoptosis by depleting mitochondrial glutathione (31) and can induce hepatocyte pyroptosis/necrosis by generating cholesterol crystals within lipid droplets (32). Importantly, concentrations of saturated fatty acid (e.g., palmitic acid), ceramide, LPC, or free cholesterol are increased in the livers of human subjects with NASH (27, 33, 34) and of animals with NASH (35), suggesting that free saturated fatty acids and other lipid metabolites as mentioned above contribute to the development and progression of NASH. Numerous studies suggest that therapeutic approaches to inhibit hepatic lipid accumulation, lipid-induced oxidative stress, and lipotoxicity-mediated cell death are promising strategies for treatment of NAFLD/NASH (36, 37). Phase 1, 2a, 2b, and 3 trials are being undertaken to test peroxisome proliferator activated receptor alpha/delta (PPARα/δ) activator (Elafibranor/GFT505; Clinical Trials NCT02704403), liver X receptor alpha (LXRα) inhibitor (Oltipraz; NCT02068339), acetyl-CoA carboxylase (ACC) inhibitors (GS-0976; NCT02856555 and PF-05221304; NCT03248882), stearoyl-CoA desaturase 1 (SCD1) inhibitor (Aramchol; NCT02279524), diacylglycerol acyltransferase 1 (DGAT1) inhibitor (Pradigastat/LCQ908; NCT01811472), and DGAT2 inhibitor (PF-06865571; NCT03513588) as targeting strategies to reduce hepatic steatosis in NASH patients via enhancement of β-oxidation or inhibition of fatty acid/TG synthesis. Natural antioxidants such as vitamin E and resveratrol have been used (or are being investigated) as therapeutic compounds to attenuate oxidative stress (36, 38). Caspase inhibitor (Emricasan/IDN-6556; NCT02686762) and apoptosis signaling kinase-1 (ASK-1) inhibitor (Selonsertib/GS-4997; NCT03053050), which target hepatic cell death, are also being evaluated in phase 2b and 3 studies of NASH patients, respectively.

In particular, ASK1 inhibitor can reduce hepatic steatosis, inflammation, and fibrosis (39, 40). In the unstimulated state, inactive ASK1 forms a high-molecular protein complex through homotypic interaction between two adjacent carboxy-terminal coiled-coil domains and through binding of its N-terminal region to thioredoxin (TRX). In an oxidative stressed state, ASK1 dissociated from TRX is homo-oligomerized in association with TNF receptor-associated factor (TRAF) and is subsequently auto-phosphorylated on threonine residue Thr838, which ultimately leads to the formation of active ASK1 signalosome. Then, this complex phosphorylates and activates p38 and JNK, resulting in increase of hepatocyte injury via the BAX-caspase pathway, impairment of hepatic insulin resistance via Ser307 phosphorylation of insulin receptor substrate, increase of hepatic inflammation via pro-inflammatory cytokine/chemokine production, and increase of hepatic fibrosis via collagen production (39, 40). Recent emerging evidence suggests that several ASK1-interacting proteins such as Dickkopf-3 (DKK3), CASP8 and FADD-like apoptosis regulator (CFLAR, known as cFlip), and TNFα-induced protein 3 (TNFAIP3, known as A20) represent attractive therapeutic targets for the prevention and treatment of NAFLD/NASH (41–43). Further understanding of molecular mechanisms underlying the regulation of ASK1 activity will provide opportunities to identify novel therapeutic targets and to develop promising therapeutic compounds for treatment of NAFLD/NASH.

#### Hepatocyte-derived secretory cytokines, damage-associated molecular patterns (DAMPs), and extracellular vesicles

Several studies suggest that hepatocyte-derived factors (or molecules) can act on non-parenchymal cells such as Kupffer cells and HSCs, which can in turn contribute to the progression of NASH (Figure 1) (44–46). In response to various stimuli such as excessive lipids and lipopolysaccharide (LPS), hepatocytes can directly produce chemokines [chemokine (C-C motif) ligand 2 (CCL2)] and pro-inflammatory cytokines (TNFα, IL-6 and IL-1β), although the amount of chemokines/cytokines released from hepatocytes is lower compared to that released from non-parenchymal cells. Subsequently, increased chemokine/cytokine production results in infiltration of macrophages into the liver and activation of Kupffer cells/HSCs (44, 46). In addition, sublethal or lethal hepatocellular injuries (apoptosis, necrosis, necroptosis, or pyroptosis) trigger signals for NASH promotion through releases of inflammatory cytokines, damage-associated molecular patterns (DAMPs) such as high mobility group box 1(HMGB1), ATP, uric acid, or mitochondrial DNA, and extracellular vesicles (18, 44, 47). DAMPs can cause hepatic inflammation and fibrosis by activating Kupffer cells and HSCs via paracrine or endocrine actions (44, 47).

It has been reported that circulating levels of extracellular vesicles such as microvesicles (microparticles) and exosomes are increased in NASH mouse models (48, 49) and patients with NASH (50). Extracellular vesicles released from hepatocytes treated with lipotoxic fatty acids can act as messenger signals mediating intercellular communication between hepatocytes and non-parenchymal cells, which contributes to aggravation of inflammation and fibrosis in NASH (48, 49, 51). For example, saturated fatty acids stimulate the release of extracellular vesicles including vanin-1, a surface cargo protein found on hepatocytes, leading to increases in chemotaxis and migration of endothelial cells (48). Consequently, enhanced angiogenesis promotes recruitment of inflammatory cells and fibrogenesis of HSCs, contributing to the progression of NASH. In addition, fatty acid-induced release of exosomes containing microRNAs (miR-128-3p) contributes to the activation of HSCs in fibrosis (49). Palmitic acid and LPC also induce the release of extracellular vesicles from hepatocytes via the death receptor 5 (DR5) signaling pathway or rho-associated coiled-coil-containing protein kinase 1 (ROCK1) (51). In this process, TRAIL-bearing extracellular vesicles activate inflammatory responses in macrophages via the DR5 signaling pathway in a receptor-interacting serine/threonine-protein kinase 1 (RIPK1)-dependent manner (51). Furthermore, mass spectrometry analysis demonstrated the presence of many DAMPs in extracellular vesicles released from hepatocytes (52). All these findings suggest that extracellular vesicles play key roles in intercellular communication between different cell types in the liver and in the development and progression of NASH. Due to the importance of extracellular vesicles in NASH pathogenesis, strategies to block the release of extracellular vesicles and to inhibit specific molecules (or cargos) within extracellular vesicles have been considered as therapeutic interventions for NASH.

#### Kupffer cells/infiltrating macrophages, inflammation, and NASH

In addition to hepatocyte-derived factors, lipotoxic fatty acids such as palmitic acid and ceramide can activate Kupffer cells and subsequently promote the production of pro-inflammatory cytokines (TNFα and IL-6) (Figure 1) (53). Kupffer cells also secrete several chemokines such as CCL2, chemokine (C-X-C motif) ligand 16 (CXCL16), and CXCL1/2/8 to recruit peripheral macrophages, natural killer T cells, and neutrophils, respectively (Figure 1) (54–56). Consequently, Kupffer cells and recruited innate immune cells can coordinately aggravate inflammation in the liver. Moreover, several cytokines such as TGFβ, lectin galactose binding soluble 3 (LGALS3, known as Galectin-3), platelet-derived growth factor (PDGF), and TNFα/IL-1β produced from Kupffer cells/infiltrating macrophages are able to activate quiescent HSCs and increase proliferation or survival of HSCs (Figure 1) (57–60). In addition, palmitic acid-induced TNFα and IL-1β in infiltrating macrophages can cause lipid accumulation and insulin resistance in hepatocytes (61).

Inflammasome is a cytoplasmic multiprotein complex that is activated by two distinct signals: the first signal for upregulation of inflammasome-related genes [pro-IL-1β, NLR family pyrin domain containing 3 (NLRP3), and Caspase 1] and a second signal for functional inflammasome assembly and activation of Caspase 1 (62). Inflammasome-mediated IL-1β secretion is initiated by activation of toll-like receptors (TLR) as a priming signal and stimulated by diverse second signals such as DAMPs, pathogen-associated molecular patterns (PAMPs) (e.g., pore-forming toxin), and toxic lipids (e.g., palmitic acids and cholesterol crystals) (63). NLRP3 inflammasome-dependent IL-1β secretion from activated Kupffer cells/infiltrating macrophages and hepatocytes plays a crucial role in progression of NAFLD/NASH (64, 65). Mice with global overexpression of mutant NLRP3 (constitutive active) showed severe hepatocyte pyroptotic cell death and severe hepatic inflammation/fibrosis, while myeloid cell (Kupffer cells)-specific mutant NLRP3-overexpressing mice exhibited mild hepatic inflammation/fibrosis in the absence of hepatocyte pyroptotsis (64), suggesting that both Kupffer cells and hepatocytes are important in NLRP3 inflammation-mediated liver injury. In parallel, NLRP3 knockout mice showed improved hepatic injury, inflammation, and fibrosis in the liver after a choline-deficient amino acid-defined (CDAA) diet feeding (65). Furthermore, treatment with MCC950 (CP-456773), a small molecule NLRP3 inhibitor, attenuates liver inflammation and fibrosis in mice fed a methionine-choline-deficient (MCD) diet (66). IL-1β knockout mice also displayed reduced steatohepatitis and hepatic fibrosis after an atherogenic cholesterol-rich diet feeding (67). In contrast, IL-1β receptor antagonist (IL-1Ra) knockout mice showed aggravated hepatic steatosis, inflammation, and fibrosis after the same diet feeding (68). All these results suggest that inhibition of NLRP3 or IL-1β could be an attractive therapeutic strategy for treatment of NALFD/NASH.

Due to the crucial role of Kupffer cell/infiltrating macrophages-derived cytokines/chemokines in steatosis, inflammation, and fibrosis, functional inhibition of these secretory proteins is considered as a promising therapeutic approach for treatment of NASH. An orally available drug capable of inhibiting recruitment of monocyte/macrophage and activation of HSCs, dual chemokine (C-C motif) receptor 2 (CCR2)/CCR5 antagonist (Cenicriviroc: NCT03028740) is currently being investigated in a phase 3 clinical trial of human NASH patients with liver fibrosis. An oral inhibitor (BI1467335/PXS-4728A; NCT03166735) of amine oxidase copper-containing 3 (AOC3) is being evaluated in a phase 2a NASH study as an anti-inflammatory compound to block the recruitment of immune cells in the liver. Galectin-3, a Kupffer cell/macrophage-derived lectin, is required for TGFβ-mediated activation of HSCs (59), and an inhibitor of Galectin 3 (GR-MD-02; NCT02462967) is also being evaluated as an anti-fibrotic drug in a phase 2b trial in NASH patients.

#### HSCs, fibrosis, and NASH

HSCs, the major source of type I collagen in the liver, play a crucial role in NASH-related fibrosis (69). Hepatocyte-derived factors (cytokines, DAMPs, or extracellular vesicles) and Kupffer/macrophage-released cytokines/chemokines trigger signaling cascades to transform “quiescent” HSCs into “activated” HSCs (myofibroblast-like cells) (69). Consequently, activated HSCs increase the secretion of collagen and other extracellular matrix proteins, leading to fibrotic scarring and ultimately chronic fibrosis in the liver (69). Thus, one of the potential strategies to inhibit NASH-related fibrosis is to suppress accumulation of extracellular matrix proteins including collagen or to directly inhibit HSC activation. Lysyl oxidase-like 2 (LOXL2), an enzyme promoting collagen cross-linking, is upregulated in the livers of animals with fibrosis (70) and of diabetic patients with NAFLD (71) and is essential in hepatic fibrogenesis (72). Phase 2b clinical studies in NASH patients using an antibody against LOXL2 (Simtuzumab/GS-6624; NCT01672866 and NCT01672879) were recently terminated. Simtuzumab had no effect on hepatic fibrosis in NASH patients with bridging fibrosis or cirrhosis (73). Heat shock protein 47 (HSP47) is a molecular chaperone which plays a crucial role in the maturation and secretion of collagen. While vitamin A-coupled lipid nanoparticle (BMS-986263/ND-LO2-s0201) containing siRNA against HSP47 is being investigated in preclinical stage for treatment of NASH, efficacy of BMS-986263 in human fibrosis patients (NCT02227459) and human cirrhosis patients (NCT03420768) are being evaluated in phase 1 and 2 clinical trials, respectively.

### Inter-organ crosstalk and NASH

Recent knowledge regarding the roles and contributions of adipose tissue and intestine in the pathogenesis of NASH have been extensively highlighted in previous reviews (5, 6). Here, we briefly describe the relationships between the adipose tissue-liver axis or intestine-liver axis and NASH.

#### Adipose tissue-liver axis and NASH

Obesity-associated insulin resistance in adipose tissue contributes to the development and progression of hepatic steatosis and NASH (5). As mentioned previously, impaired suppression of insulin-mediated lipolysis in white adipose tissue (WAT) leads to hepatic steatosis through increased fatty acid uptake of hepatocytes (Figure 1). In addition, inflammation in WAT systemically affects hepatic inflammation (74). Lean mice transplanted with visceral adipose tissue from obese mice exhibited elevated infiltration of neutrophils and macrophages in the liver and ultimately suffered from aggravated liver damage after NASH diet feeding (74). However, transplantation of visceral fat from obese mice with depletion of adipose tissue macrophage (ATM) by treatment with clodronate liposomes did not cause hepatic inflammation in lean mice (74), suggesting that ATM directly contributes to hepatic inflammation and NASH progression. Furthermore, adipokines (such as adiponectin and leptin) secreted from WAT have reported to affect lipid accumulation, inflammation, and fibrosis in the liver (Figure 1) (75).

In addition to the role of WAT, BAT (brown adipose tissue) is associated with the development and progression of NAFLD. A couple of previous papers suggest that transplantation of BAT alleviates hepatic steatosis in HFD-fed obese mice and leptin-deficient ob/ob mice (76, 77). Conversely, treatment with propranolol, a β-adrenergic receptor antagonist, worsens liver injury in mice fed a half-methionine and choline-deficient diet supplemented with ethionine (HMCDE) due to increased hepatocyte cell death (78). Furthermore, a recent paper suggests that thermoneutral housing exacerbates HFD-driven NAFLD in mice, which is related to reduced activation of BAT, although additional events (augmented intestinal permeability, dysbiosis of the microbiome, and altered immune responsiveness caused by decreased norepinephrine/corticosterone) could also contribute to these phenotypes (79). Despite the presence of severe steatosis and inflammation in HFD-fed mice housed at a thermoneutral temperature, hepatic fibrosis did not develop in these mice (79). Thus, it will be interesting to investigate whether activation of thermogenic adipocytes ameliorates the development and progression of NASH in mice fed NASH diets such as MCD diet, high-fat/high-cholesterol diet, or high-fat/high-fructose diet.

#### Intestine-liver axis and NASH

Growing evidence suggests that the intestine-liver axis plays a crucial role in the maintenance of metabolic homeostasis, and that its impairment is an important causal factor in the pathogenesis of diverse liver diseases such as obesity-related steatosis, NAFLD/NASH, and liver cancer (80, 81). Feeding of HFD or NASH diets causes impairment of intestinal barriers, dysbiosis of the microbiota, and alterations of intestinal immunity, leading to increased translocation of bacteria or bacterial products into the systemic circulation (82, 83). Consequently, bacteria or bacterial products are able to reach the liver through the portal vein. In the liver, conserved motifs/structures of bacteria and bacterial products (PAMPs) are recognized by pathogen recognition receptors (PPRs) of various cell types (hepatocytes, Kupffer cells/infiltrating macrophages, and HSCs). In particular, PAMPs such as LPS, peptidoglycan, and bacterial DNA stimulate multiple signaling cascades via interactions with PAMP-specific TLRs and NOD-like receptors (NLRs), leading to hepatic steatosis, inflammation, and fibrosis in the liver (Figure 1) (6, 84).

Emerging evidence suggests that lipid intermediates derived from the intestine trigger the development of NAFLD/NASH and insulin resistance (85, 86). In mice fed diets supplemented with palmitic acid or palm oils, concentrations of ceramide were increased in the intestine and serum/plasma (87). Ceramide derived from the intestines of mice fed HFD systemically causes ER/mitochondrial stresses and increases fatty acid synthesis in hepatocytes, which leads to hepatic lipid accumulation, hepatic cell death, and inflammation (Figure 1) (85, 86). Intriguingly, these phenotypes are attenuated in intestine-specific hypoxia-inducible factor 2α (HIF2α)- or farnesoid X receptor (FXR)-knockout mice which showed decreased intestinal ceramide level due to reduced expression of HIF2α- or FXR-target genes involved in ceramide synthesis (85, 86). Furthermore, intestinal ceramide production and hepatic steatosis are attenuated in HFD-fed mice treated with antibiotics (85), suggesting that HFD-induced alterations of the microbiota contribute to increased intestinal ceramide production and hepatic lipid accumulation. In particular, taurocholic acid (TCA) and tauro-β-muricholic acid (T-β-MCA) produced from the liver competitively act as agonist and antagonist for FXR signaling in intestinal epithelial cells, respectively (85). In mice fed HFD, T-β-MCA is converted to MCA by bile salt hydrolase (BSH), a microbial enzyme, which results in aggravated hepatic steatosis and hepatic injury due to increased intestinal ceramide production via activation of TCA-mediated agonistic action for FXR and inhibition of T-β-MCA-mediated antagonistic action (85). In HFD-fed mice treated with antibiotics, however, accumulated T-β-MCA inhibits intestinal FXR signaling and subsequently suppresses intestinal ceramide synthesis, leading to improvements in hepatic steatosis and hepatic injury (85). In addition, hormones derived from the intestine influence the development and progression of NAFLD/NASH (88), which will be discussed in next section. In view of the importance of impaired intestinal barrier-induced penetration of microbial products and microbial dysbiosis-induced changes of intestinal signaling in the pathogenesis of NAFLD/NASH, therapeutic modulation of the intestine-liver axis represents an attractive strategy for treatment of NAFLD/NASH. A phase 2a clinical study using IMM-124E (composed of anti-LPS antibody and glycosphingolipid adjuvants) is currently underway in human NASH patients (NCT02316717). Further understanding of intestine-liver interactions will help identify novel therapeutic targets and molecules to prevent and treat NAFLD/NASH.

## Hormone-based therapeutic approaches for treatment of NASH

Based on the “multiple-parallel hit” model of the pathogenesis of NASH, therapeutic approaches such as reduction of steatosis, blockade of hepatic cell death, suppression of hepatic immune cells, and inhibition of fibrogenic action of HSCs are considered as attractive strategies for treatment of NASH. Since hormones systemically influence diverse tissues (or cell types) in the body, they can have effects on multiple steps in the pathogenesis of NASH. Thus, hormone-based therapy is an attractive strategy for treatment of NASH. In this section, we briefly review current hormone-based NASH therapies.

### Fibroblast growth factor 19 (FGF19)-based NASH therapy

FGF19 (FGF15 in rodents) is a postprandial endocrine hormone that is produced in the intestine by bile acid-induced FXR activation (89), and that plays a key role in the regulation of bile acid and lipid metabolism in the liver (89). FGF19 also inhibits gluconeogenesis and stimulates hepatic glycogen and protein synthesis via insulin-independent action (90). In addition to its physiological effects, therapeutic administration of FGF19 or genetic overexpression of FGF19 also have pharmacological effects such as decreased hepatosteatosis/adiposity and improved insulin sensitivity via enhancement of β-oxidation/thermogenesis, inhibition of lipogenesis, or amelioration of lipotoxicity-induced ER stress (91–93). In contrast, HFD-fed FGF15 knockout mice showed increased adiposity or exacerbated ER stress and hepatosteatosis (93). Recent emerging studies suggest that FGF19 ameliorates muscle wasting via direct action on skeletal muscle (94) and corrects type 1 diabetes via inhibition of the hypothalamic–pituitary–adrenal axis (95). Intriguingly, circulating FGF19 level is decreased in human NAFLD/NASH subjects, and hepatic response to FGF19 is impaired in human NAFLD subjects with insulin resistance (96, 97). Therapeutic administration of an engineered FGF19 (NGM282/M70, a nontumorigenic FGF19 variant) eliminates lipotoxicity and bile acid toxicity, leading to improvements of steatohepatitis and fibrosis in a NASH mouse model (98). However, endogenous FGF15 deletion had no effect on steatosis, inflammation, or fibrosis in mice fed HFD for 6 months (99). In a phase 2a clinical study (NCT02443116), treatment of human NASH patients with NGM282 resulted in reduced hepatic steatosis and decreased markers of hepatic inflammation/fibrosis with acceptable safety (100).

### FGF21-based NASH therapy

FGF21 is expressed as an endocrine hormone predominantly in the liver and other metabolic tissues such as adipose tissue, muscle, and pancreas. FGF21 plays physiologically important roles in the regulation of glucose/lipid metabolism and maintenance of energy balance in response to changes in nutritional status such as starvation (101, 102) and environmental stimuli such as cold exposure or exercise (103, 104). FGF21 can exert beneficial effects on obesity and related metabolic diseases (105, 106). Pharmacological treatment with FGF21 or overexpression of FGF21 improves diet-induced obesity and insulin resistance by enhancing insulin-mediated glucose uptake and β-oxidation/thermogenesis (105, 106). Furthermore, FGF21 ameliorates obesity-induced ER stress, increased serum levels of liver enzymes, and insulin resistance (107). Emerging evidence suggests that FGF21 is also implicated in the pathogenesis and treatment of NASH (108, 109). FGF21 is increased in the livers of NASH animal models (108) and of human patients with NASH (109). Therapeutic treatment with FGF21 or overexpression of FGF21 causes improvements of MCD diet-induced steatosis, inflammation, and fibrosis in mice by reducing hepatic lipotoxicity and increasing β-oxidation (108). Moreover, treatment with FGF21 suppresses PDGF-induced activation of HSCs *in vitro* (110), implying that direct anti-fibrogenic action of FGF21 in HSCs may contribute to FGF21-induced improvement of fibrosis *in vivo*. In line with preclinical studies, a recent phase 2a clinical study using a pegylated analog of FGF21 (PEG-FGF21, BMS-986036) suggests reduction of steatosis and improvement of markers of fibrosis and liver injury in human NASH patients (111). Phase 2b clinical studies (NCT03486899 and NCT03486912) to evaluate the safety and efficacy of BMS-986036 in human NASH patients with severe stage 3 fibrosis or cirrhosis were recently started.

### Glucagon-like peptide 1 (GLP-1)-based NASH therapy

GLP-1 is a proglucagon-derived hormone that is secreted from the intestine in response to changes in nutrients (112). GLP-1 plays a crucial role in the regulation of glucose metabolism by enhancing insulin release, suppressing glucagon secretion, and inhibiting gastric emptying (112). Furthermore, pharmacological treatment with GLP-1 leads to improvements of diet-induced obesity and insulin resistance by suppression of food intake and enhancement of thermogenesis (113, 114). Seven synthetic GLP-1 receptor agonists (exenatide, exenatide long-acting release, liraglutide, albiglutide, dulaglutide, lixisenatide, and semaglutide) are approved and available for treatment of type 2 diabetes. Intriguingly, GLP-1 analogs also ameliorate not only hepatic steatosis, but also hepatic inflammation and fibrosis in mice (115). Furthermore, in a phase 2 clinical study (NCT01237119) for efficacy of liraglutide in human NASH patients, patients receiving liraglutide for 48 weeks showed significant reductions of hepatic ballooning, steatosis, and serum alanine aminotransferase (ALT) level compared to patients who received placebo (116). Liraglutide-induced reduction of hepatic lipid accumulation is probably due to decreased hepatic *de novo* fatty acid synthesis (117). A phase 2 clinical study using semaglutide in human NASH patients (NCT02970942) is currently underway.

In line with the therapeutic effect of GLP-1, pharmacological treatment with sitagliptin or linagliptin, inhibitors of dipeptidylpeptidase 4 (DPP4), an enzyme that degrades GLP-1, improves hepatic steatosis, inflammation, and fibrosis in NASH mouse models (118, 119). Furthermore, vildagliptin, another DPP4 inhibitor, ameliorates serum liver enzyme levels and hepatic steatosis in human NAFLD patients with dyslipidemia (120). In contrast, sitagliptin treatment for 24 weeks had no beneficial effects on serum liver enzyme levels, fatty liver, or fibrosis in human NASH or NAFLD patients (NCT01260246 and NCT01963845) (121, 122). Further large-scale studies are needed to evaluate the efficacy and clinical importance of DDP4 inhibitor for treatment of NAFLD/NASH.

Glucagon/GLP-1 receptor dual agonists and glucagon/glucose-dependent insulinotropic polypeptide (GIP)/GLP-1 receptor triple agonists are attractive therapeutic agents to treat NAFLD/NASH as well as obesity-related diabetes (123, 124). Some preclinical studies have shown improvement of obesity-related metabolic deterioration in mice treated with glucagon/GLP-1 receptor dual agonist (125) or glucagon/GIP/GLP-1 receptor triple agonist (126). Pharmacological treatment with a pegylated analog of oxyntomodulin (PEG-OXM, G49), a natural agonist of the glucagon/GLP-1 receptor, improves steatohepatitis in MCD diet-fed mice, probably due to reductions of ER/mitochondrial stresses and hepatocyte apoptosis (127). Treatment with glucagon/GIP/GLP-1 receptor triple agonists also leads to significant improvement of steatohepatitis in female mice fed high-fat/high-sucrose diets and also in male mice, albeit to a lesser extent (128). While several phase 1 or 2 clinical studies using glucagon/GLP-1 receptor dual agonists and glucagon/GIP/GLP-1 receptor triple agonists are being conducted in human obese diabetic patients (123, 124), clinical studies using glucagon/GLP-1 receptor dual agonist (SAR425899; NCT03437720) and glucagon/GIP/GLP-1 receptor triple agonist (HM15211) for NASH patients have recently been registered.

### Growth differentiation factor 15 (GDF15)-based NASH therapy

GDF15, an endocrine hormone belonging to the TGFβ superfamily, is ubiquitously expressed in various tissues, with the highest levels in the liver, placenta, and macrophages (129–131). Numerous studies suggest that GDF15 is induced by diverse stress stimuli such as fatty acids, ER/mitochondrial stressors, and LPS (132–134), and that serum GDF15 level is increased in human subjects with diseases such as cardiovascular disease, chronic kidney disease, obesity, diabetes, and cancer (135, 136). Intriguingly, recent emerging evidence suggests that GDF15 exerts beneficial effects on obesity-related insulin resistance in mice and monkeys through the suppression of food intake via GDNF family receptor α-like (GFRAL)-dependent anorexic action (137–140). GDF15 also stimulates oxidative metabolism in macrophages or metabolic tissues such as adipose tissue and liver, leading to the improvement of insulin resistance and hepatic steatosis in obese mice (133, 141). In addition to its anti-obesity and anti-diabetic effects, GDF15 has been implicated in the development and progression of NAFLD/NASH (142, 143). Serum GDF15 level is elevated in NASH animal models (143) and in human subjects with NASH (142, 143). In two dietary NASH models using MCD and amylin liver NASH (AMLN) diets, GDF15 knockout mice showed deteriorated steatohepatitis and fibrosis in the liver, while GDF15 transgenic mice were resistant to diet-induced NASH phenotypes (143). Furthermore, treatment with recombinant GDF15 ameliorates lipid accumulation, inflammation, and fibrosis in mice caused by alcohol feeding (144), suggesting that GDF15 is a promising therapeutic candidate to treat alcoholic steatohepatitis (ASH) as well as NASH. While preclinical studies using GDF15 analogs have been conducted, clinical studies aimed at treating obesity-related type 2 diabetes or NAFLD/NASH have yet to be performed.

## Discussion

NAFLD/NASH is a public health problem worldwide, but there are no therapeutic drugs approved for its treatment. Numerous preclinical animal models based on genetic or dietary manipulation with relevance to human NAFLD/NASH have been developed to explore the mechanisms underlying the development of NAFLD/NASH and identify novel targets for its treatment (145, 146). The studies conducted using these models have made important steps forward in our understanding of the pathogenesis of NASH and in the discovery of potential therapeutic candidates. As discussed above, sophisticated communication between parenchymal and non-parenchymal cells in the liver or between the liver and other organs contributes to the development and progression of NAFLD/NASH (Figure 1). Thus, multiple components (hepatic lipid accumulation, oxidative stress, ER/mitochondrial stress, hepatocyte cell death, hepatocyte-released DAMPs/extracellular vesicles, Kupffer cell activation, inflammatory cell recruiting, HSC activation, insulin resistance, adipose tissue inflammation, and microbiota dysbiosis) are attractive therapeutic targets to treat NAFLD/NASH. In this review, we summarize preclinical or clinical therapeutic efficacies of several pharmacological agents targeting these multiple components of NASH. Pharmacological agents under development for treatment of NAFLD/NASH (including some candidates not discussed in this review) are listed in Table 1. Given the value of a “multiple-parallel model” in the pathogenesis of NASH, monotherapy with a single agent targeting multiple components (e.g., BMS-986036/PEG-FGF21 for targeting insulin resistance, hepatic lipid accumulation, oxidative stress, and ER stress) or combined therapy with agents targeting a single component (e.g., ASK1 inhibitor [Selonsertib] and ACC inhibitor [GS-0976]) will be efficacious therapeutic approaches for treatment of NASH, in addition to monotherapy with an agent targeting a single component. Thus, further understanding of NASH pathogenesis and preclinical/clinical studies to evaluate the efficacy of candidate agents will accelerate novel therapeutic innovations for treatment of NAFLD/NASH and related metabolic disorders.

**Table 1:** Pharmacological agents under development for treatment of NAFLD/NASH.

**Drugs**	**Target of Action**	**Company**	**Highest developmental stage/clinical trial identifier**
**BILE ACID (BA) METABOLISM-RELATED AGENTS (MONOTHERAPY)**			
Obeticholic acid/OCA (INT-747)	FXR agonist	Intercept Pharmaceuticals	Phase 3 /NCT03439254, NCT02548351
Px-104	FXR agonist	Phenex Pharmaceuticals	Phase 2a (discontinued) /NCT01999101
Tropifexor (LJN452)	non-BA FXR agonist	Novartis	Phase 2a/NCT02855164
LMB763	non-BA FXR agonist	Novartis	Phase 2a/NCT02913105
EDP-305	non-BA FXR agonist	Enanta Pharmaceuticals	Phase 2a/NCT03421431
GS-9674	FXR agonist	Gilead Sciences	Phase 2/NCT02854605
INT-767	FXR/TGR5 dual agonist	Intercept Pharmaceuticals	Pre-clinical phase
Volixibat (SHP626)	IBAT inhibitor	Shire Pharmaceuticals	Phase 2/NCT02787304
**LIPID METABOLISM-RELATED AGENTS (MONOTHERAPY)**			
Elafibranor (GFT505)	PPARα/δ activator	Genfit	Phase 3/NCT02704403
Saroglitazar	PPARα/γ activator	Zydus Discovery	Phase 2/NCT03061721
IVA337	PPARα/δ/γ activator	Inventiva Pharma	Phase 2/NCT03008070
Oltipraz	LXRα inhibitor	PharmaKing	Phase 3/NCT02068339
GS-0976	ACC inhibitor	Gilead Sciences	Phase 2a/NCT02856555
PF-05221304	ACC inhibitor	Pfizer	Phase 2a /NCT03248882
Gemcabene	ACC/ApoC-III inhibitor	Gemphire Therapeutics /Pfizer	Phase 2a/NCT03436420
Aramchol	SCD1 inhibitor	Galmed Pharmaceuticals	Phase 2b/NCT02279524
Pradigastat (LCQ908)	DGAT1 inhibitor	Novartis	Phase 2a/NCT01811472
PF-06865571	DGAT2 inhibitor	Pfizer	Phase 1/NCT03513588
MGL-3196	TRβ receptor agonist	Madrigal Pharmaceuticals	Phase 2/NCT02912260
VK2809	TRβ receptor agonist	Viking Therapeutics	Phase 2a/NCT02927184
**GLUCOSE/FRUCTOSE METABOLISM-RELATED AGENTS (MONOTHERAPY)**			
LIK066	SGLT1/2 inhibitor	Novartis	Phase 2a/NCT03205150
PF-06835919	KHK inhibitor	Pfizer	Phase 2a/NCT03256526
**LIPOTOXIC STRESS AND CELL DEATH-RELATED AGENTS (MONOTHERAPY)**			
Emricasan (IDN-6556)	Caspase inhibitor	Conatus/Novartis	Phase 2b/NCT02686762
Selonsertib (GS-4997)	ASK-1 inhibitor	Gilead Sciences	Phase 3/NCT03053050
**MITOCHONDRIAL METABOLISM-RELATED AGENT (MONOTHERAPY)**			
MSDC-0602	mTOT modulator/MPC inhibitor	Cirius Therapeutics	Phase 2b/NCT0278444
**EXTRACELLULAR VESICLES-RELATED AGENT (MONOTHERAPY)**			
RG-125 (AZD4076)	Anti-miR targeting microRNA−103/107	Regulus/Astrazenenka	Phase 1 (discontinued)/NCT02612662
**INFLAMMATION AND INFLAMMASOME-RELATED AGENTS (MONOTHERAPY)**			
MCC950 (CP-456773)	NLRP3 inhibitor	Pfizer	Pre-clinical phase
Cenicriviroc	CCR2/5 dual antagonist	Allergan	Phase 3/NCT03028740
BI1467335 (PXS-4728A)	AOC3 inhibitor	Boehringer Ingelheim/Pharmaxis	Phase 2a/NCT03166735
GRI-0621	Natural killer T cell inhibitor	GRI Bio	Phase 2a/NCT02949375
Tipelukast (MN-001)	LT/PDE/5-LO inhibitor	MediciNova	Phase 2a/NCT02681055
Nalmafene (JKB-121)	TLR4 antagonist	TaiwanJ Pharmaceuticals	Phase 2/NCT02442687
CF102	A3AR agonist	Can-Fite Biopharma	Phase 2/NCT02927314
DS102 (AF-102)	15-HEPE	DS Biopharma/Afimmune	Phase 2a/NCT02941549
**FIBROSIS-RELATED AGENTS (MONOTHERAPY)**			
GR-MD-02	Galectin-3 inhibitor	Galectin Therapeutics	Phase 2b/NCT02462967
Simtuzumab(GS-6624)	LOXL2 inhibitor	Gilead Sciences	Phase 2b (discontinued)/NCT01672866, NCT01672879
BMS-986263(ND-LO2-s0201)	HSP47 siRNA	Nitto Denko/BMS	Pre-clinical phase
**INTESTINE AND MICROBIOTA-RELATED AGENTS (MONOTHERAPY)**			
IMM-124E	anti-LPS antibody and glycosphingolipid adjuvants	Immuron	Phase 2a/NCT02316717
Solithromycin	Macrolide antibiotic	Cempra Inc	Phase 2/NCT02510599
**INSULIN RESISTANCE-RELATED AGENTS (MONOTHERAPY)**			
RO5093151	11β-HSD1 inhibitor	Hoffmann-La Roche	Phase 1/NCT01277094.
NS-0200	Leucine/Metformin/Sildenafil	NuSirt Biopharma	Phase 2/NCT02546609
**HORMONE-RELATED AGENTS (MONOTHERAPY)**			
NGM282	FGF19 analog	NGM BIO	Phase 2a/NCT02443116
BMS-986036(PEG-FGF21)	FGF21 analog	BMS	Phase 2b/NCT03486899, NCT03486912
Liraglutide	GLP-1 receptor agonist	Novo Nordisk	Phase 2/NCT01237119
Semaglutide	GLP-1 receptor agonist	Novo Nordisk	Phase 2/NCT02970942
Sitagliptin	DPP4 inhibitor	MSD	Phase 2/NCT01963845 Not applicable /NCT01260246
Vildagliptin	DPP4 inhibitor	Novartis	Not applicable
SAR425899	Glucagon/GLP-1 receptor dual agonist	Sanofi-Aventis	Phase 2/NCT03437720
G49 (PEG-OXM)	Glucagon/GLP-1 receptor dual agonist	MedImmune	Pre-clinical phase
HM15211	Glucagon/GIP/GLP-1 receptor triple agonist	Hanmi Pharmaceutical	Phase 1/Clinical trial approval (April 2018)
NGM386, NGM395	GDF15 analogs	NGM BIO	Pre-clinical phase
YH25724	GLP-1/FGF21 dual agonist	Yuhan Corporation	Pre-clinical phase
**COMBINED THERAPY**			
Tropifexor (LJN452) +Cenicriviroc (CVC)	FXR agonist + CCR2/5 dual antagonist	Novartis + Allergan	Phase 2b/NCT03517540
Selonsertib (GS-4997)+ GS-0976 + GS-9674	ASK-1 inhibitor +ACC inhibitor + FXR agonist	Gilead Sciences	Phase 2/NCT02781584, NCT03449446

^*^A3AR, A3 adenosine receptor; ACC, acetyl-CoA carboxylase; AOC3, amine oxidase copper-containing 3; ASK-1, apoptosis signaling kinase-1; CCR2/5, dual chemokine (C-C motif) receptor 2/5; DGAT, diacylglycerol acyltransferase; DPP4, dipeptidylpeptidase 4; FGF, fibroblast growth factor; FXR, farnesoid X receptor; GDF15, growth differentiation factor 15; GIP, glucose-dependent insulinotropic polypeptide; GLP-1, glucagon-like peptide-1; 15-HEPE, 15-hydroxyeicosapentaenoic acid; 11β-HSD1, 11-beta-hydroxysteroid dehydrogenase type 1; HSP47, heat shock protein 47; IBAT, ileal apical sodium-dependent bile acid transporter; KHK, ketohexokinase; LOXL2, lysyl oxidase-like 2; LPS, lipopolysaccharide; LT/PDE/5-LO, leukotriene/phosphodiesterase/5-lipoxygenase; LXR, liver X receptor; NLRP3, NLR family pyrin domain- containing 3; MPC, mitochondrial pyruvate carrier; mTOT, mitochondrial target of thiazolidinedione; PPAR, peroxisome proliferator activated receptor; SCD1, stearoyl-CoA desaturase 1; SGLT, sodium/glucose cotransporter; TGR5, takeda G protein-coupled receptor 5; TLR4, toll-like receptor 4; TR, thyroid hormone receptor.

## Author contributions

All authors listed have made a substantial, direct and intellectual contribution to the work, and approved it for publication.

### Conflict of interest statement

The authors declare that the research was conducted in the absence of any commercial or financial relationships that could be construed as a potential conflict of interest.
